# Supplementary material for: Human stereoEEG recordings reveal network dynamics of decision-making in a rule-switching task
Source: Nat Commun. 2020 Jun 17;11:3075. doi: 10.1038/s41467-020-16854-w (PMC7300004; doi:10.1038/s41467-020-16854-w)
Supplement: Supplementary file 1 — Supplementary Information [file 41467_2020_16854_MOESM1_ESM.pdf]

Supplementary Information

Human stereoEEG recordings reveal network dynamics of decision-making in a rule-switching task

M ter Wal, *et al*.

Correspondence: m.j.terwal@bham.ac.uk

Content:

Supplementary Tables	2
Supplementary Figures	6
Supplementary References	21

Supplementary Tables

Patient ID	Gender	Age (yrs)	School years	Hemi-sphere	Number of electrodes	MR	Post-Surgical Pathology	AEDs
P01	F	39	13	R	16	NEG	FCD IIa	CBZ, PHT
P02	M	26	13	R	13	NEG	gliosis	PRI, CBZ, CLB
P03	M	19	10	L	12	NEG	gliosis	LCS, PER, TPM
P04	M	28	13	L	12	HS	n.a.	CBZ, LTG, CLB
P05	M	40	8	L	13	NEG	gliosis	OXC, TPM
P06	M	44	8	L	17	NEG	gliosis	CBZ, LTG, CLB

Supplementary Table 1. Demographical, clinical, and anatomical variables for all tested patients. Abbreviations: In the Gender column: F means female, M means male. In the MR column, NEG means negative, HS indicates hippocampal sclerosis. In Post-Surgical Pathology, n.a. (not applicable) indicates that the patient did not undergo surgical intervention (generally because thermo-coagulations were successful), FCD indicates that pathology was confirmed or indicates a focal cortical dysplasia (Type IIa is mostly invisible in MR examination, Type IIb is evident). Anti-epileptic drugs (AEDs) include CBZ (Carbamazepine), CLB (Clobazam), LCS (Lacosamide), LTG (Lamotrigine), OXC (Oxcarbazepine), PER (Perampanel), PHT (Phenytoin), PRI (Primidone), TPM (Topiramate).

Patient ID	Semantic fluency	Naming	Visual exploration	Executive function (attention matrices)	Face recognition
P01	39/2	22	n.a.	56	45
P02	35/2	22	34	59	**39**
P03	34/1	23	34	57	45
P04	51/4	24	31	42	45
P05	31/2	24	35	58	41
P06	29/1	24	34	42	47

Supplementary Table 2. Neuropsychological variables of all tested patients. Neuropsychological tests focused on the evaluation of the patient skills in language (production, comprehension, and reading), verbal memory, visuospatial memory, visual exploration, executive and attentional functions, visual perception, and abstract reasoning. Among them, we considered of particular relevance five items indexing skills relevant to the required tasks, which are presented here. For semantic fluency[1] the overall score is followed by the ranked index; where a value greater or equal to 2 indicates normal function, a value of 1 indicates a subclinical abnormality, and a value of 0 indicates a pathological dysfunction. The second item, naming, was extracted from the Boston Naming Test[2] and a score below 20 is considered pathological. For visual exploration[3] a score below 30 is considered pathological. For the fourth item, attentional matrices[1], normal scores exceed 40. Lastly, face recognition was evaluated with the Benton Facial Recognition Test[4] for which normal values range from 41 to 54. All values are within normal range, except one just outside normal range in face recognition (bold), and two borderline scores in semantic fluency (/1).

Patient ID	# gray matter leads	# gray matter leads in EZ
P01	119	3
P02	115	30
P03	101	8
P04	95	18
P05	92	27
P06	141	10

Supplementary Table 3. Number of leads in white and gray matter for each patient, as well as the number of gray matter leads in the Epileptic Zone (EZ) as defined by the clinical team.

Patient ID	# correct trials	# timed-out trials	# incorrect trials	# rejected trial-lead pairs - % (range of # rej. trials per lead)
P01	782	0	18	507 - 0.53% (0-25)
P02	788	0	12	939 - 1.0% (0-55)
P03	441	19	20	394 - 0.81% (0-23)
P04	435	13	32	685 - 1.5% (0-27)
P05	416	38	26	178 - 0.40% (0-23)
P06	263	155	62	327 - 0.48% (0-28)

Supplementary Table 4. The number of correct and incorrect trials for each patient. The table also gives the number of trial-channel pairs that were rejected during visual inspection due to epileptic activity, both in absolute numbers and as a percentage of total number of trial-lead pairs. In brackets, the range of rejected trial numbers per lead is given.

Area	1. Subsampled to 50% of trials – 10 iterations										2. Subsampled to 30% of trials – 10 iterations										3. Subsampled incongruent trials – 10 iterations									
PMm	7	6	6	6	6	6	6	6	6	6	6	6	6	5	6	6	6	6	6	6	6	6	6	6	6	6	6	6	7	5
PMv	0	0	0	1	1	0	0	0	0	0	0	0	0	0	0	1	0	0	0	0	0	0	0	0	0	0	0	0	0	0
PMd	4	3	4	1	1	3	2	3	1	1	2	1	1	3	3	1	1	1	2	2	2	0	4	1	0	0	1	1	2	1
lPFC	2	2	1	2	2	2	2	1	1	2	1	1	0	2	0	0	1	1	1	1	0	0	1	1	0	0	0	0	2	1
OP1	6	7	9	8	10	7	4	8	7	6	3	6	7	8	4	2	6	4	5	6	4	0	5	4	3	4	3	2	4	3
OP2	1	1	1	1	1	1	1	1	1	1	1	1	1	1	1	1	1	1	1	1	1	1	1	1	1	1	1	1	1	1
OP3	6	5	5	4	5	6	5	4	7	5	2	2	4	4	3	4	1	2	4	3	4	2	4	2	2	2	5	3	4	4
OP4	1	1	1	0	1	1	2	0	2	2	0	0	0	0	0	0	0	1	1	0	2	0	0	0	0	0	0	0	0	1
PF	1	0	0	0	0	1	0	0	0	0	0	0	0	0	0	0	0	0	0	0	0	0	0	0	0	0	0	0	0	0
PFcm	2	1	0	2	3	2	1	1	3	3	1	0	1	1	2	1	0	0	1	1	2	0	0	1	0	1	1	1	1	0
PFop	5	5	4	5	5	5	5	5	6	5	4	4	5	6	5	4	3	4	4	4	5	6	4	5	3	5	4	4	4	5
PFt	3	2	2	2	3	3	2	1	2	3	2	2	2	0	1	1	0	1	1	2	0	1	2	0	0	0	3	0	2	1
PCC	1	0	0	0	0	0	0	0	0	0	0	0	0	0	0	0	0	0	1	0	0	0	0	0	0	0	0	0	0	0
PGa	0	0	0	0	0	0	0	0	0	0	0	1	0	0	0	0	0	0	0	0	0	0	0	0	0	0	0	0	0	0
TL	6	3	3	2	5	5	4	3	2	5	2	2	3	2	3	5	3	4	4	2	2	4	3	2	3	2	2	4	3	3
Insula	1	1	0	0	0	0	1	0	0	1	0	0	0	0	0	1	0	0	0	0	0	0	0	0	0	0	0	0	0	0
BA44	0	0	0	0	0	0	0	0	0	0	0	0	0	0	0	0	0	0	0	0	0	0	0	0	0	0	0	0	0	0
BA45	0	0	0	0	0	0	0	0	0	1	0	0	0	0	0	0	0	0	1	0	0	0	0	0	0	0	0	0	0	0
FL	0	2	3	2	0	0	0	1	1	0	0	1	0	0	3	0	0	2	2	1	1	0	2	3	0	0	1	1	3	3
BA4	9	8	8	7	9	8	10	11	10	8	9	7	8	8	8	7	8	8	8	6	7	6	8	7	7	6	7	6	8	8
BA3a	4	3	4	3	3	3	3	3	4	3	3	3	3	3	3	3	3	3	3	3	3	4	3	3	3	3	3	4	4	3
S1	9	9	9	9	9	9	9	9	9	9	9	9	9	8	8	9	7	9	8	9	7	8	9	9	8	9	8	7	8	8

Supplementary Table 5. Raw data for the 10 subsampled classifiers shown in Figure 3 and Supplementary Figures 10 and 11. Given are the number of leads per brain area that reached significance in each of the 10 iterations for the manipulations 1-3. The averages and standard deviations for these manipulations are plotted in: 1) the gray bars in Figure 3, Supplementary Figure 10a and Supplementary Figure 11; 2) the gray bars in Supplementary Figure 10b and 3) the purple bars in Supplementary Figure 10b.

Supplementary Figures

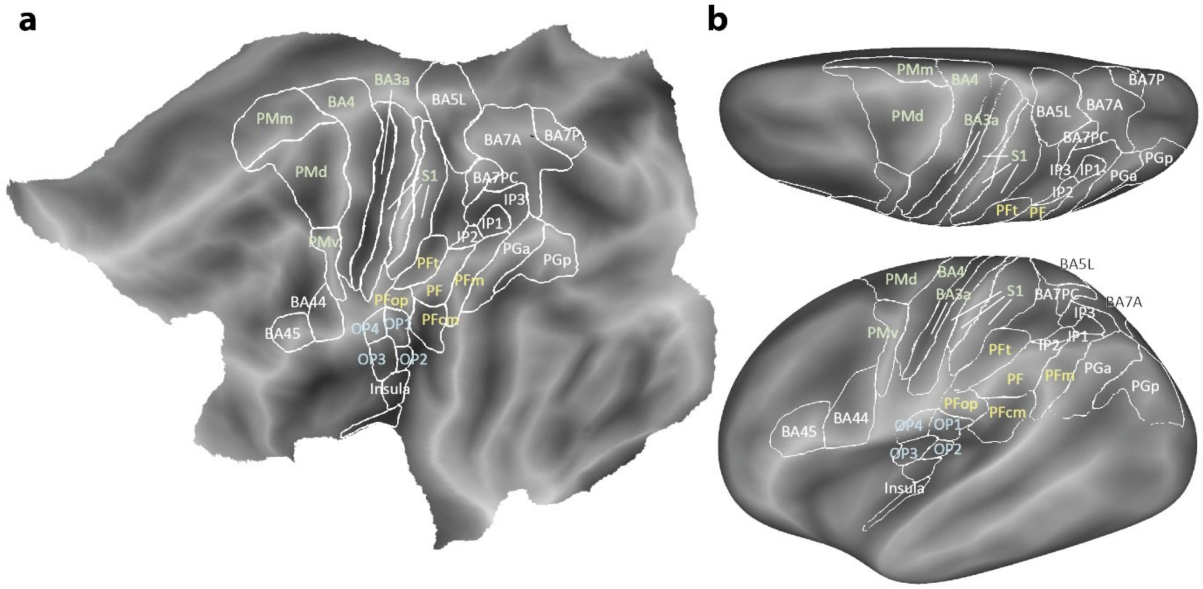

Supplementary Figure 1. Subdivision of the brain regions of interest. Flatmap (a) and dorsal and lateral views of an inflated brain surface (b) of the left hemisphere with labels for all anatomical regions from the Anatomy Toolbox[5]. Green areas are in premotor, motor and somatosensory cortex, yellow indicates areas in supramarginal gyrus, parietal operculum regions are in blue.

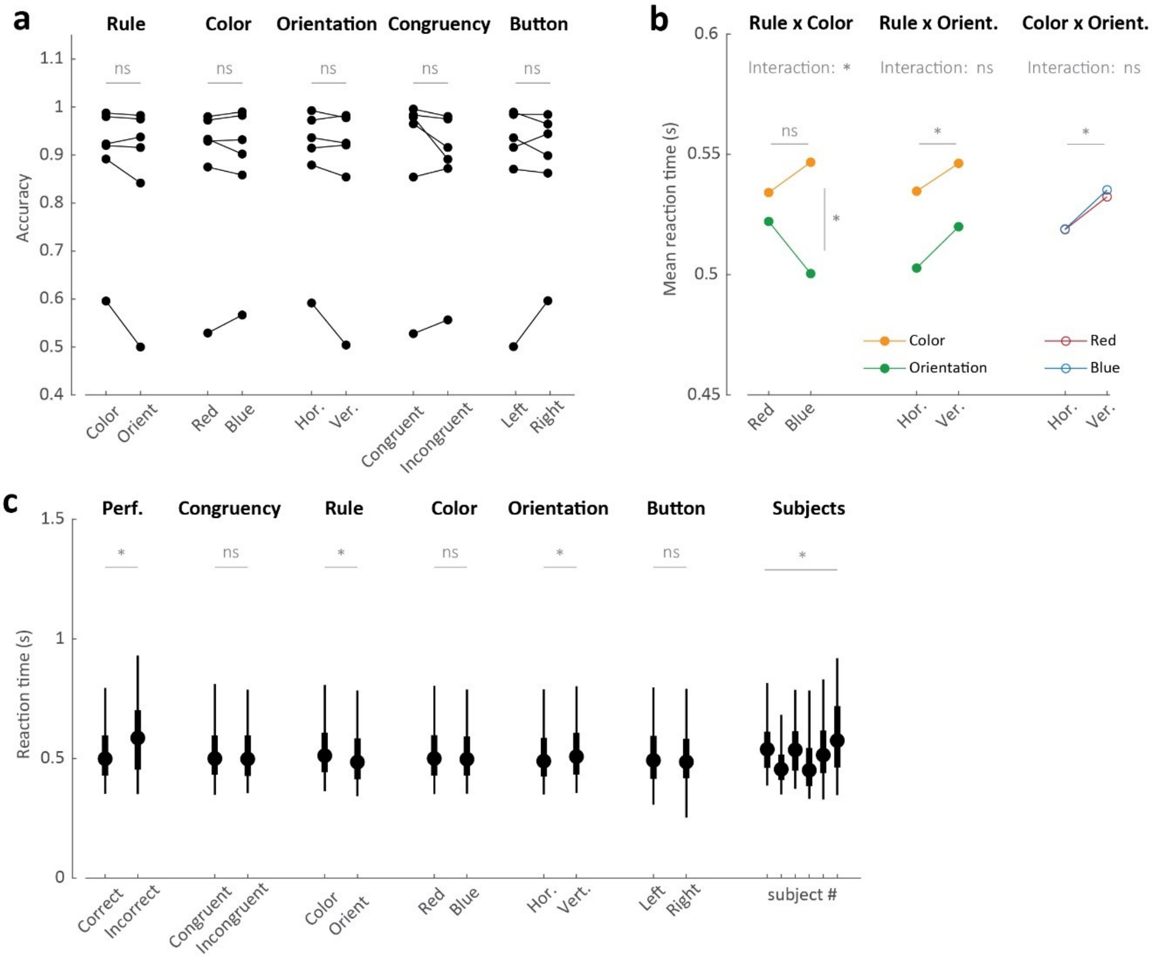

Supplementary Figure 2. Accuracy and reaction times split across the different task dimensions. a: Accuracy for individual task dimensions per subject. Connected dots represent data from one subject. Horizontal gray bars indicate the result of two-sided paired t-test with $\alpha = 0.01$; b: Interactions between reaction times for task rule, and stimulus color and orientation (N = 3125). Horizontal and vertical gray bars indicate significance of the main effects, while the significance of the interaction is indicated in gray above each column as analyzed with a 2-way ANOVA; c: Reaction time distributions for each of the task dimensions across all 6 subjects. The boxplots show the 5, 25, 50 (filled circles), 75 and 95% boundaries. Gray horizontal bars indicate significance as analyzed using two-tailed t-tests (all dimensions except subject) or one-way ANOVA (subjects), at $\alpha = 0.01$.

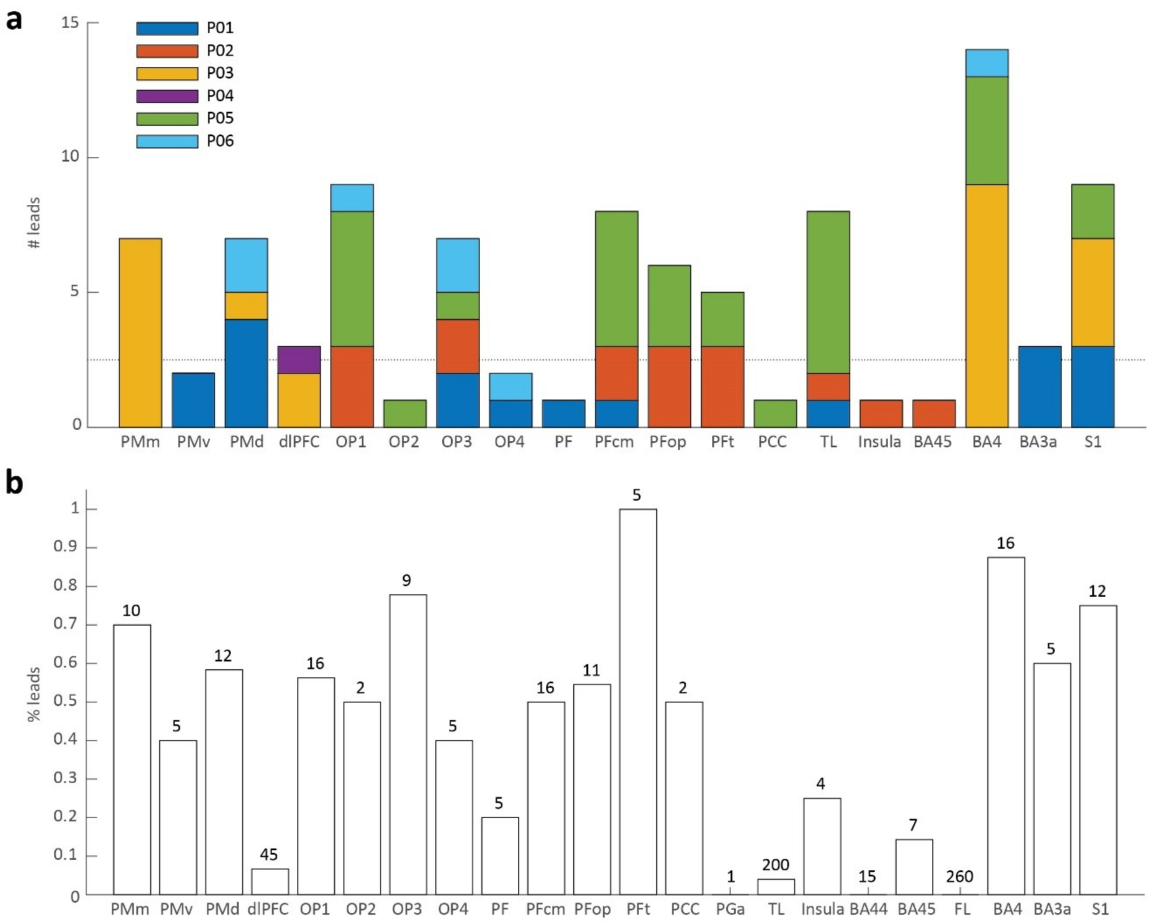

Supplementary Figure 3. The number of leads per brain area and per patient showing significant left/right classifier performance. a: The number of leads per patient, for all areas with at least one lead showing significant left/right decoding. Each color is one patient. The dotted line indicates the threshold for inclusion in the analysis in main figures 3-5; b: Leads with significant decoding as a percentage of all recorded leads in an area. The number above each bar indicates the total number of recorded leads within the area. PM stands for premotor, OP for parietal operculum, PCC for posterior cingulate cortex, TL for temporal lobe, FL for other frontal lobe leads outside of dlPFC, motor and premotor areas.

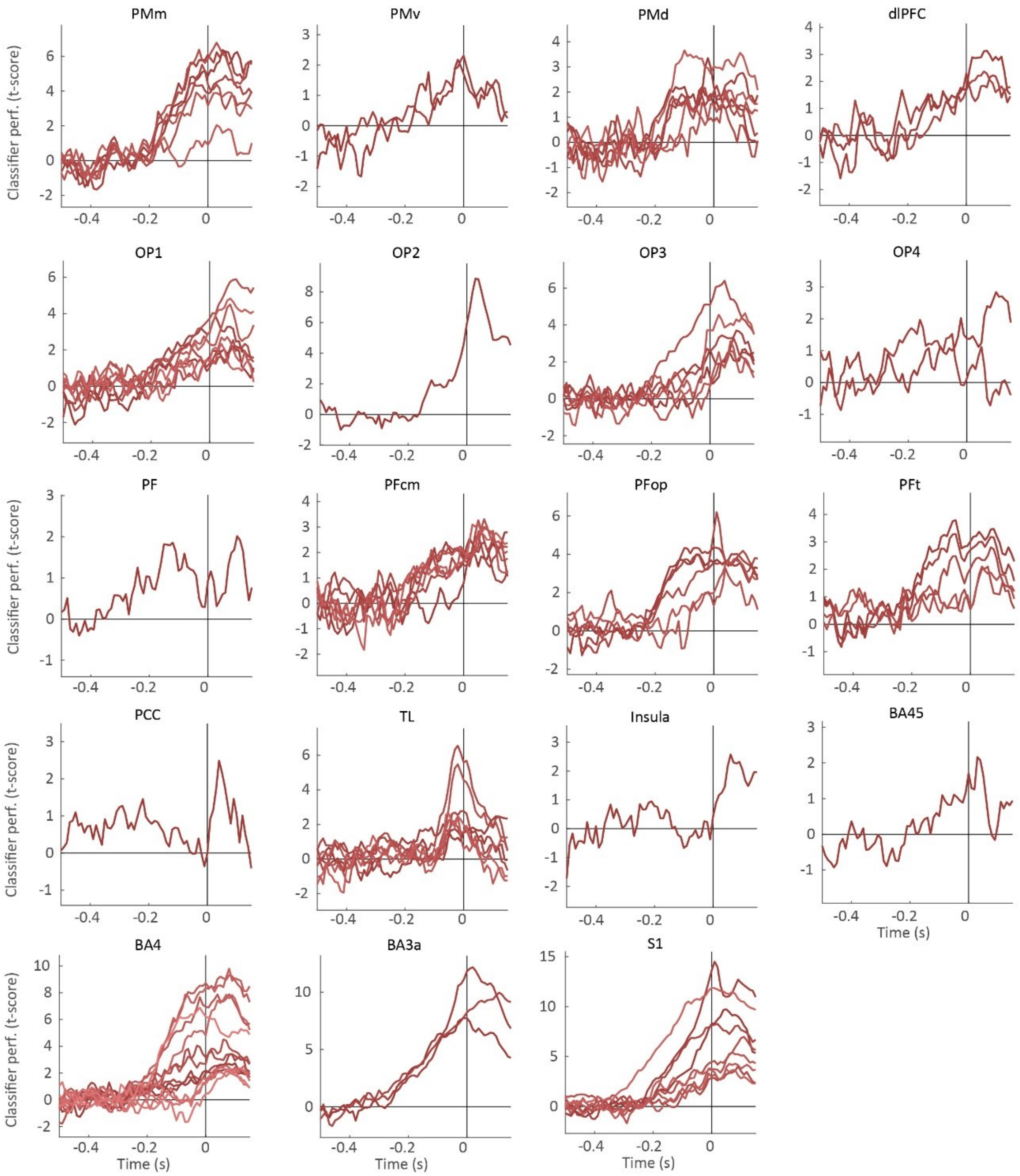

Supplementary Figure 4. Performance for the left/right classifier, for each significant lead (one line), aggregated according to brain area. The x-axes show the time leading up to the response (vertical black line). Classifier performance is given as a t-score. Note that the y-axis differs between panels.

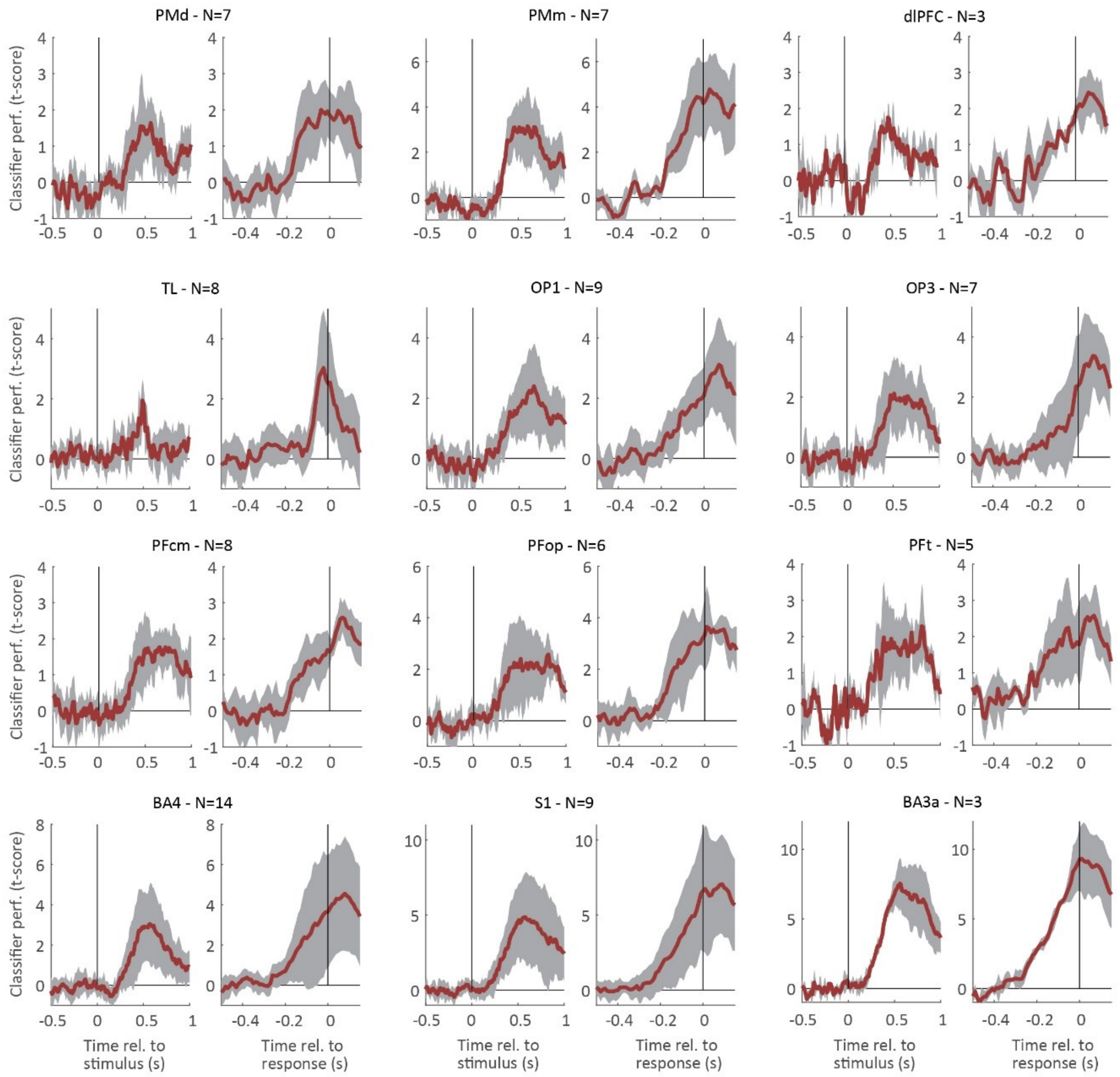

Supplementary Figure 5. Average stimulus-locked (left panels) and response-locked (right panels) classifier performance traces per brain region (for all brain regions with at least 3 significant leads). Gray areas give the standard deviation across leads. The number of leads in a region is given in the title above each panel. Classifier performance is given as a t-score. Note that the y-axis differs between brain regions.

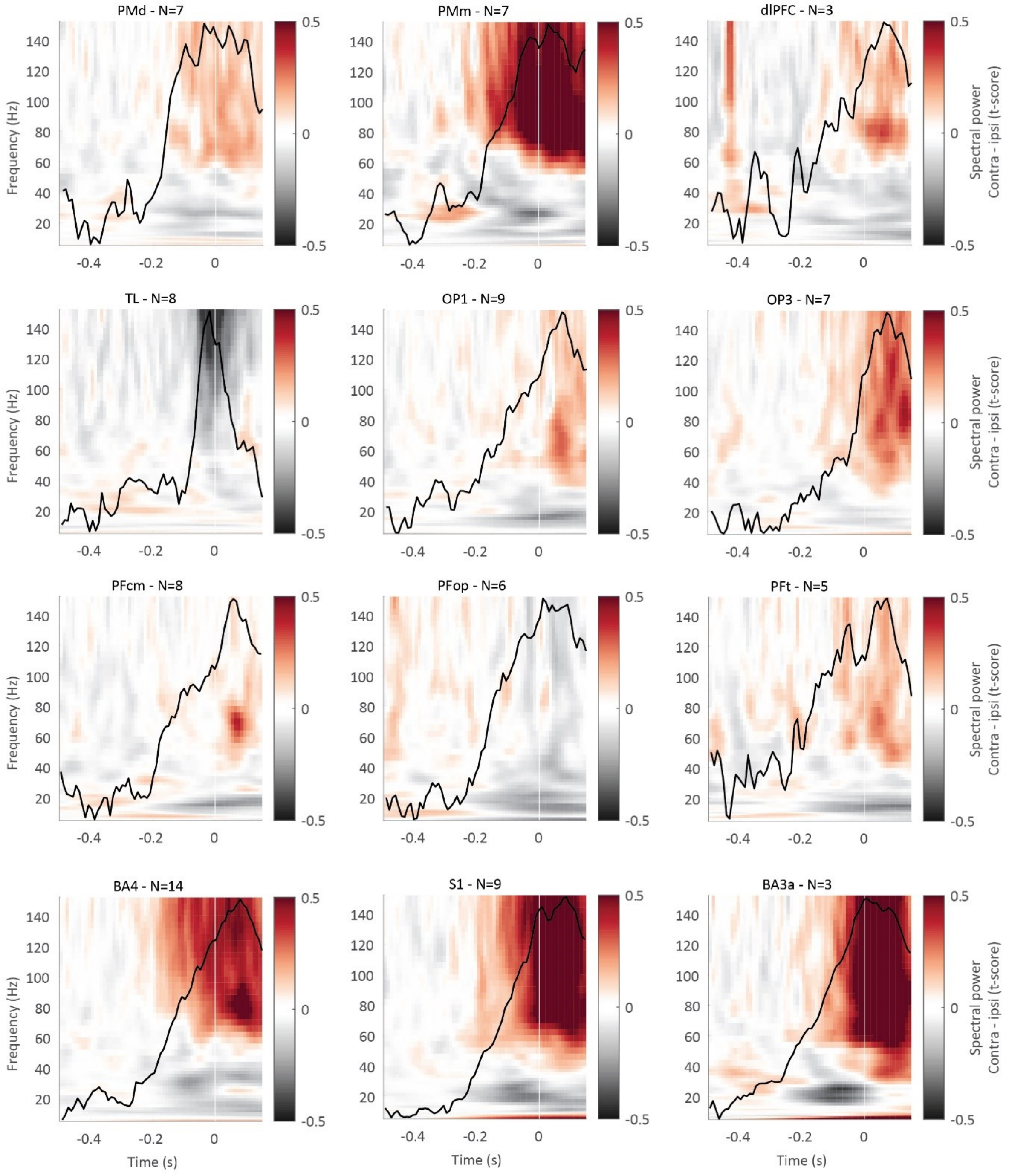

Supplementary Figure 6. Average power contrast (t-scored) between contralateral and ipsilateral button presses per brain area. The x-axes give the time leading up to the response (vertical white line). The black lines represent the normalized average classifier performance across all leads with significant left/right classification within the brain area (y-axis between 0 and 1). The number of leads N contributing to the averages is given above each panel.

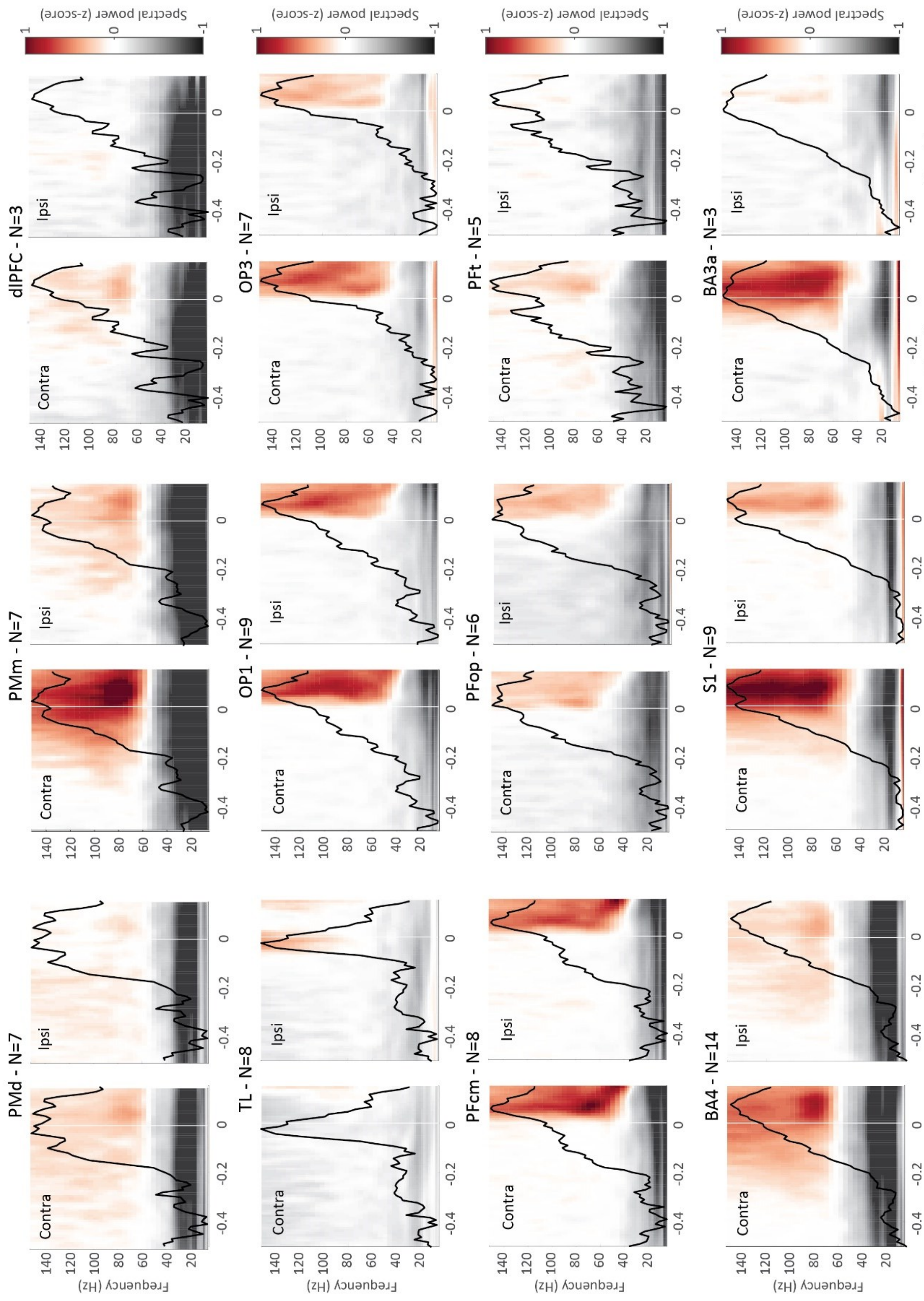

Supplementary Figure 7. Average power for contralateral and ipsilateral button presses per brain area z-scored relative to the pre-cue baseline. The x-axes give the time leading up to the response (vertical white line). The black lines represent the normalized average classifier performance across all leads with significant left/right classification within the brain area (y-axis between 0 and 1). The number of leads N contributing to the averages is given above each panel.

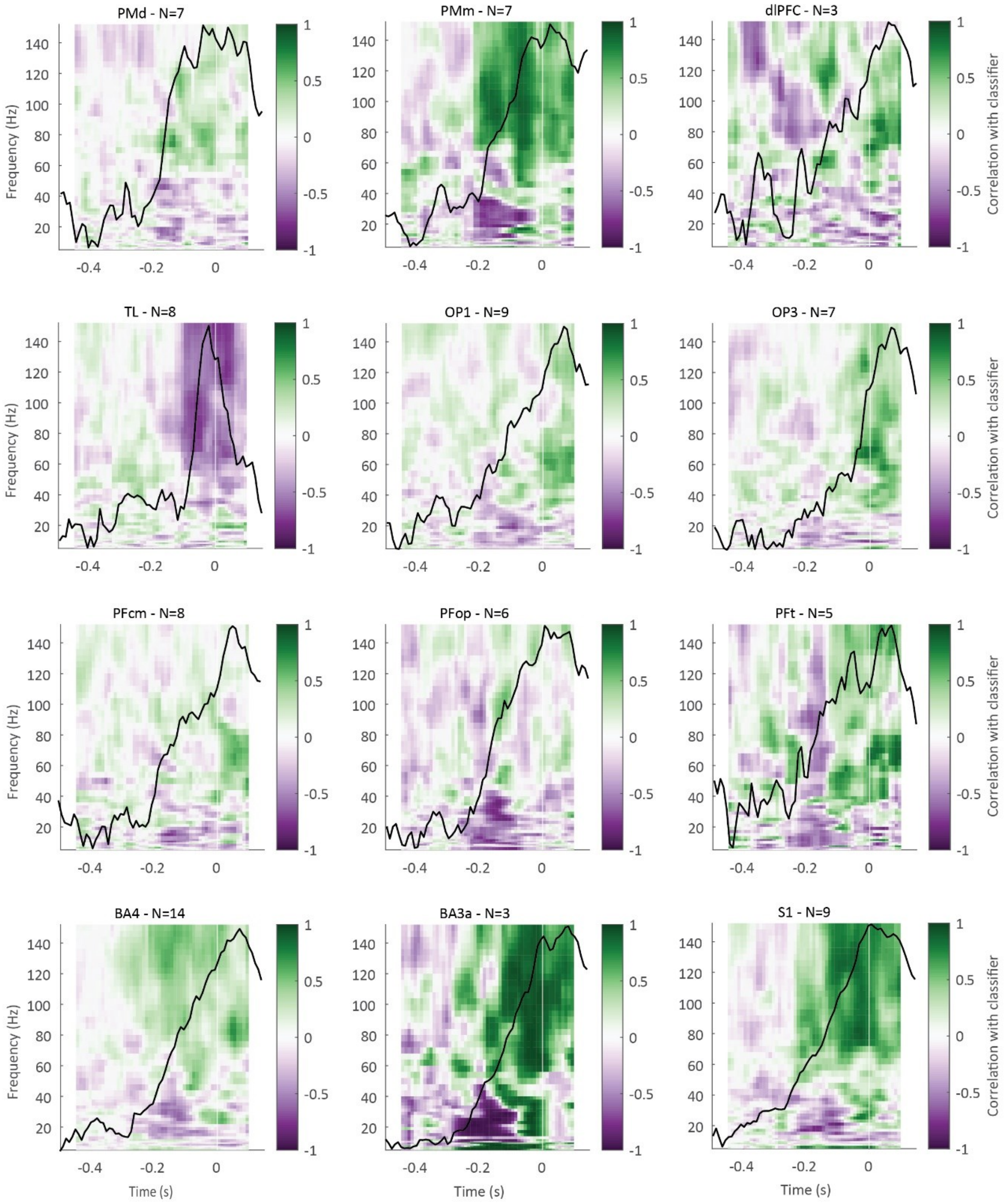

Supplementary Figure 8. Average correlation between classifier performance and power difference between contralateral and ipsilateral button presses per brain area. The x-axes give the time leading up to the response (vertical white line). The black lines represent the normalized average classifier performance for the brain area, based on all leads showing significant left/right classification (y-axis between 0 and 1). The number of leads N contributing to the correlation is given above each panel.

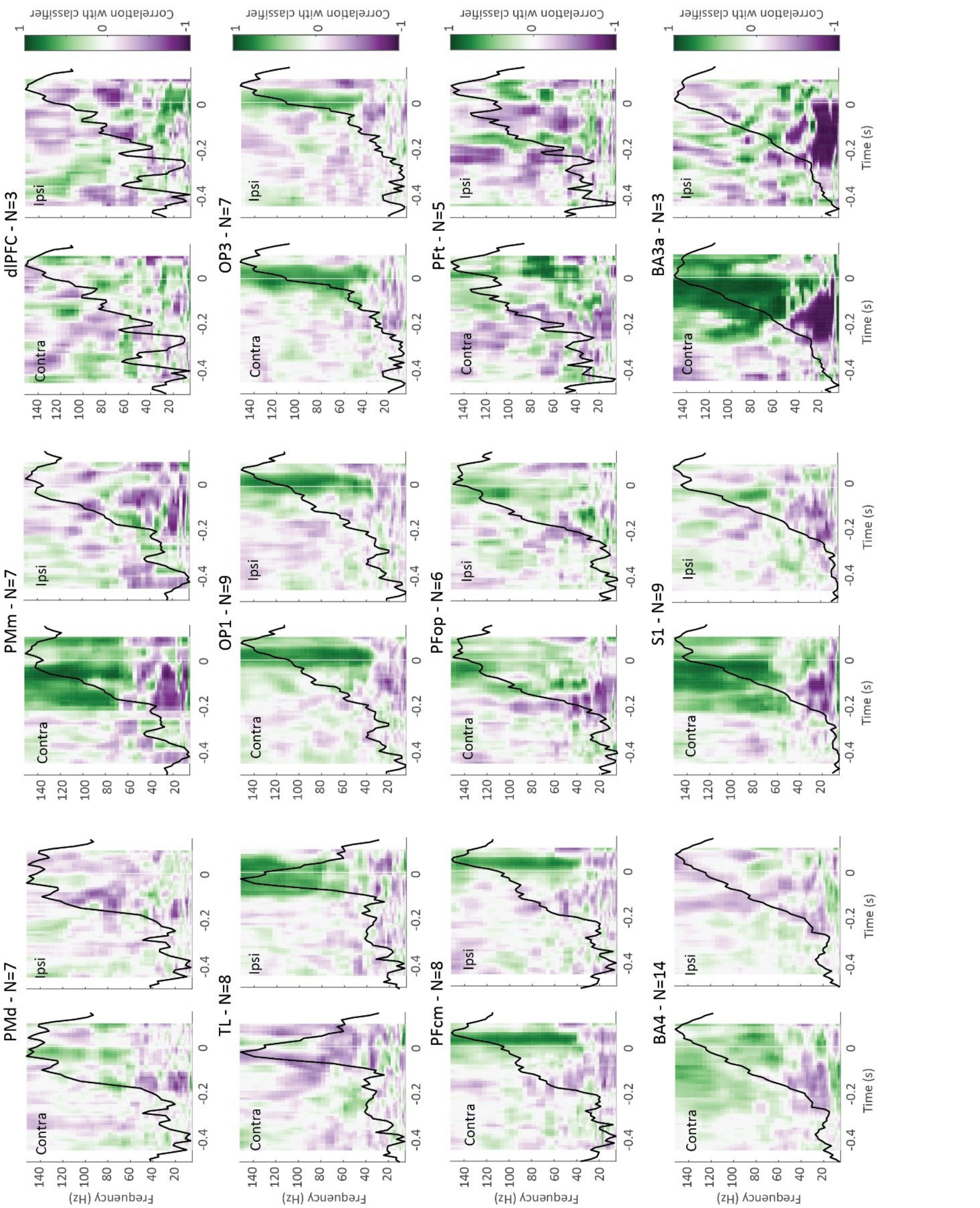

Supplementary Figure 9. Average correlation between classifier performance and power (z-scored relative to pre-cue baseline) for contralateral and ipsilateral button presses per brain area. The x-axes give the time leading up to the response (vertical white line). The black lines represent the normalized average classifier performance for the brain area, based on all leads showing significant left/right classification (y-axis between 0 and 1). The number of leads N contributing to the correlation is given above each panel.

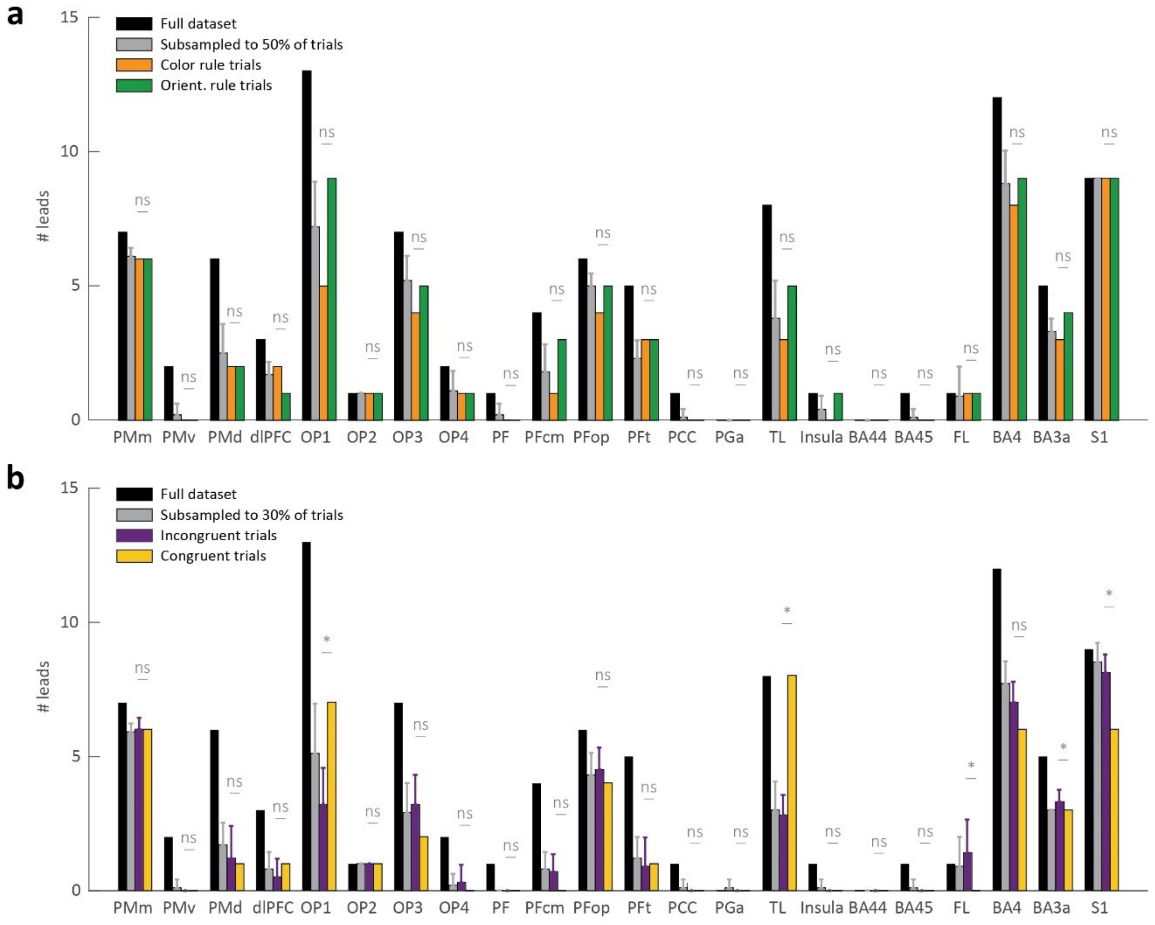

*Supplementary Figure 10. The number of leads per brain area showing significant left/right classifier performance. a: Number of significant leads for the classifiers trained on all trials (black), randomly chosen subsets with half the trial count (gray, average + standard deviation of 10 repetitions, raw data can be found in Supplementary Table 5), and color (orange) and orientation (green) rules. b: Number of significant leads for congruent (yellow) and incongruent, downsampled to match the number of congruent trials (purple, average + standard deviation of 10 repetitions, raw data in Supplementary Table 5). Black bars as in a. Gray bars indicate the number of significant leads for randomly chosen subsets containing 30% of the trials, matching the number of congruent trials. Horizontal bars indicate a one-sided Wilcoxon rank test of the difference between the number of leads for color versus orientation rule (a) and congruent versus incongruent trials (b) compared to pairs of randomly subsampled classifiers. ns means not-significant, * indicates significant at α = 0.05 (FDR corrected). PM stands for premotor, OP for parietal operculum, PCC for posterior cingulate cortex, TL for temporal lobe, FL for other frontal lobe leads outside of dlPFC, premotor and motor areas. The total number of leads per brain area can be found in Supplementary Figure 3b.*

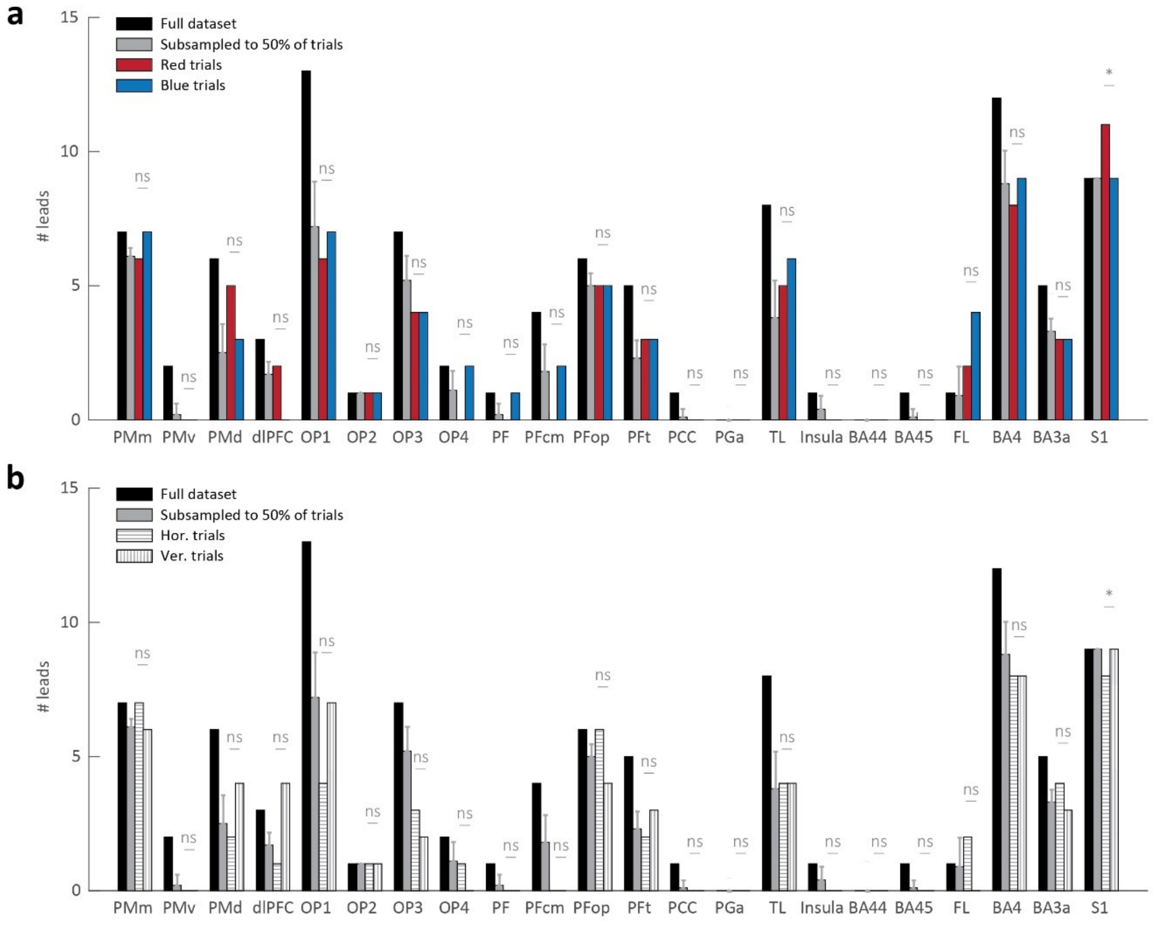

*Supplementary Figure 11. The number of leads per brain area showing significant left/right classifier performance for the classifiers trained on all trials (black), randomly chosen subsets with half the trial count (gray, average + standard deviation of 10 repetitions, raw data can be found in Supplementary Table 5), and red and blue (a) or horizontal and vertical trials only (b). Horizontal bars indicate a one-sided Wilcoxon rank test of the difference between the number of leads for blue versus red trials (a) and horizontal versus vertical trials (b) compared to pairs of randomly subsampled classifiers. ns means not-significant, * indicates significant at α = 0.05 (FDR corrected). PM stands for premotor, OP for parietal operculum, PCC for posterior cingulate cortex, TL for temporal lobe, FL for other frontal lobe leads outside of dlPFC, premotor and motor areas. The total number of leads per brain area can be found in Supplementary Figure 3b.*

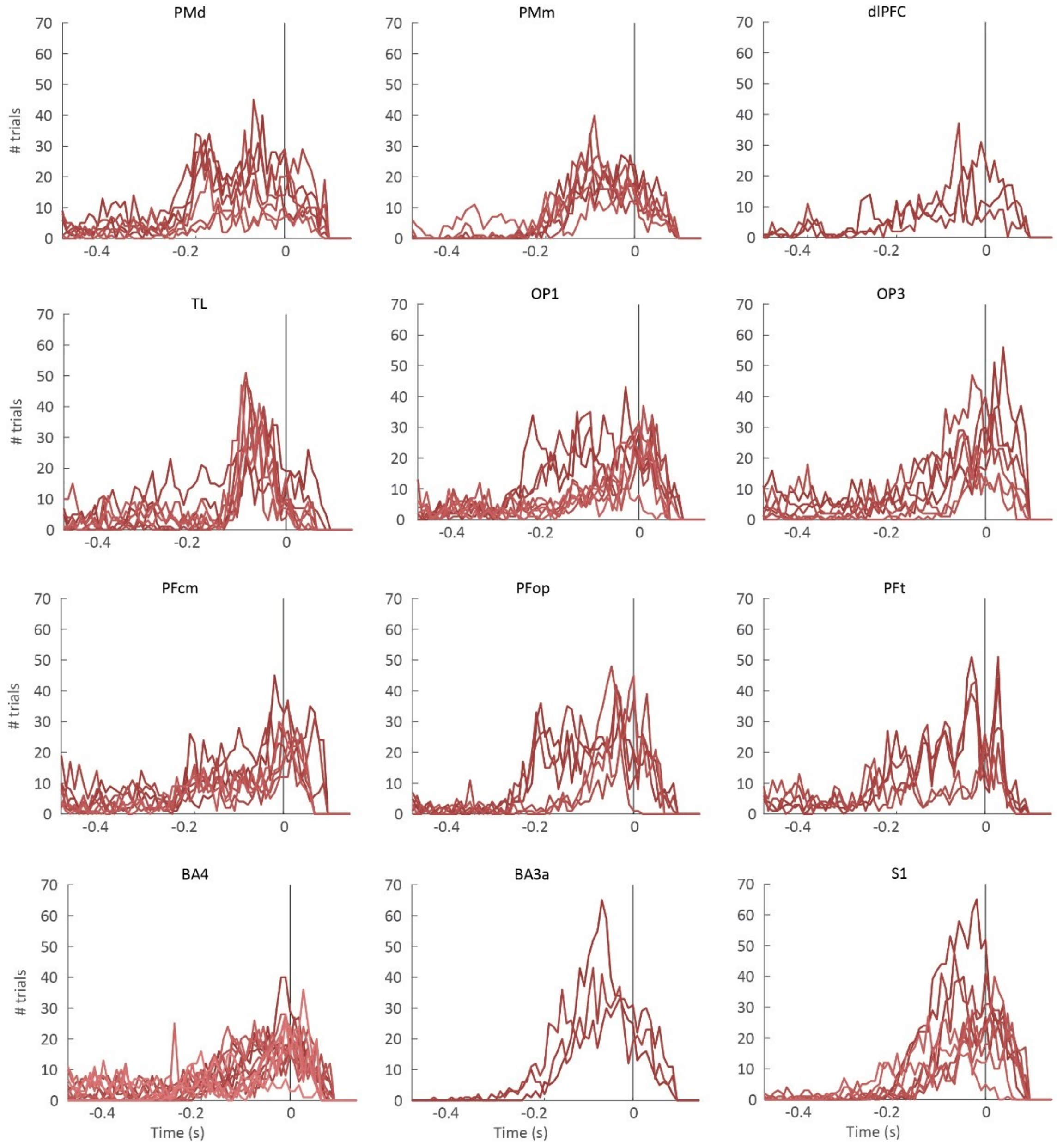

Supplementary Figure 12. Distribution of classifier onset across trials. The onset distribution is given for each significant lead (one line), organized by brain area. The x-axes show the time leading up to the response (vertical black line).

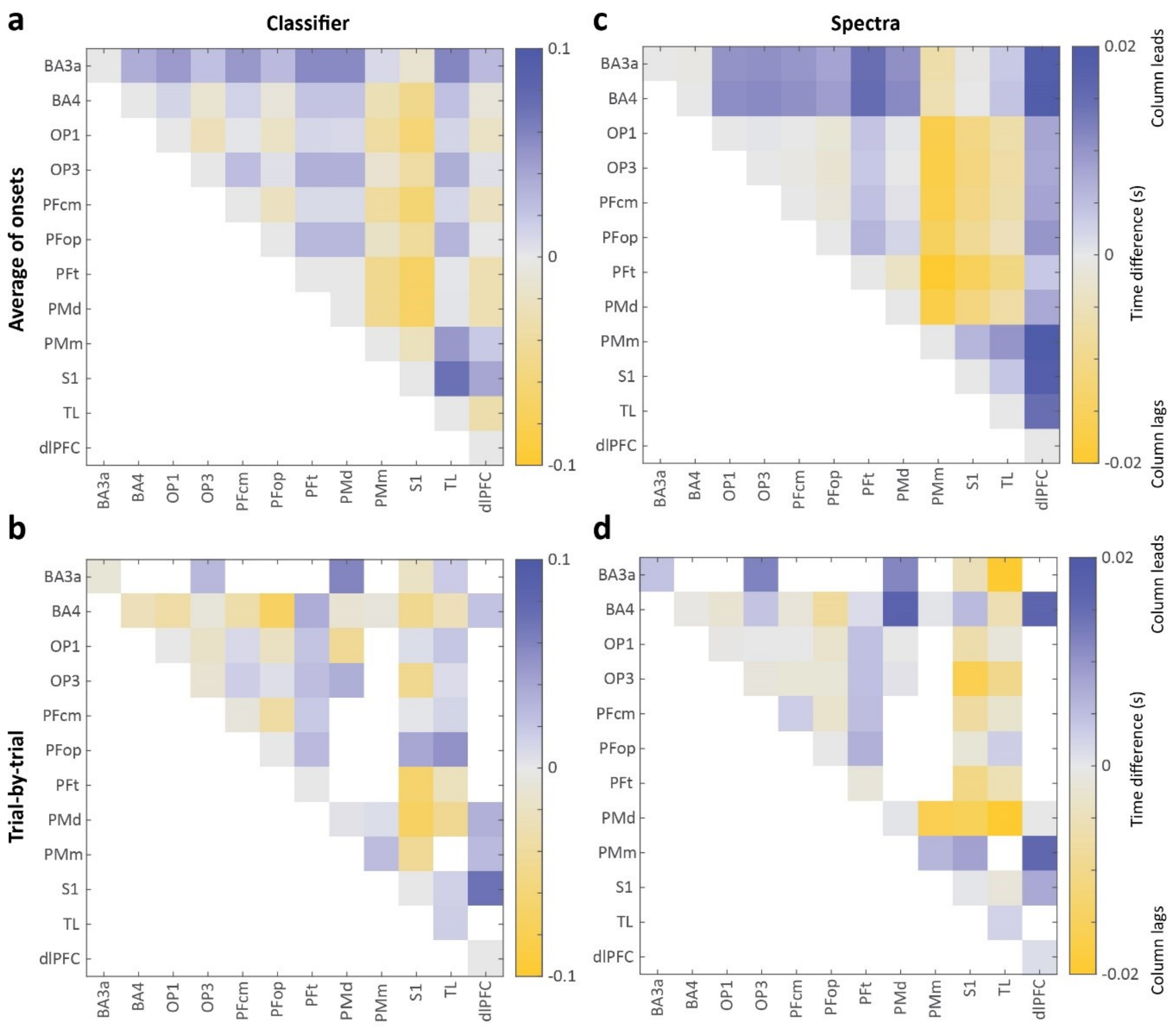

Supplementary Figure 13. Matrices showing the onset differences for all pairs of brain areas based on each of the four methods described in Figure 4, i.e. the average-onset (a & c) and trial-by-trial (b & d) approaches for both classification onset (a & b) and power contrast (c & d). Note that the scale of the color bars differs between panels. The panels a & b were also shown in Figure 4a.

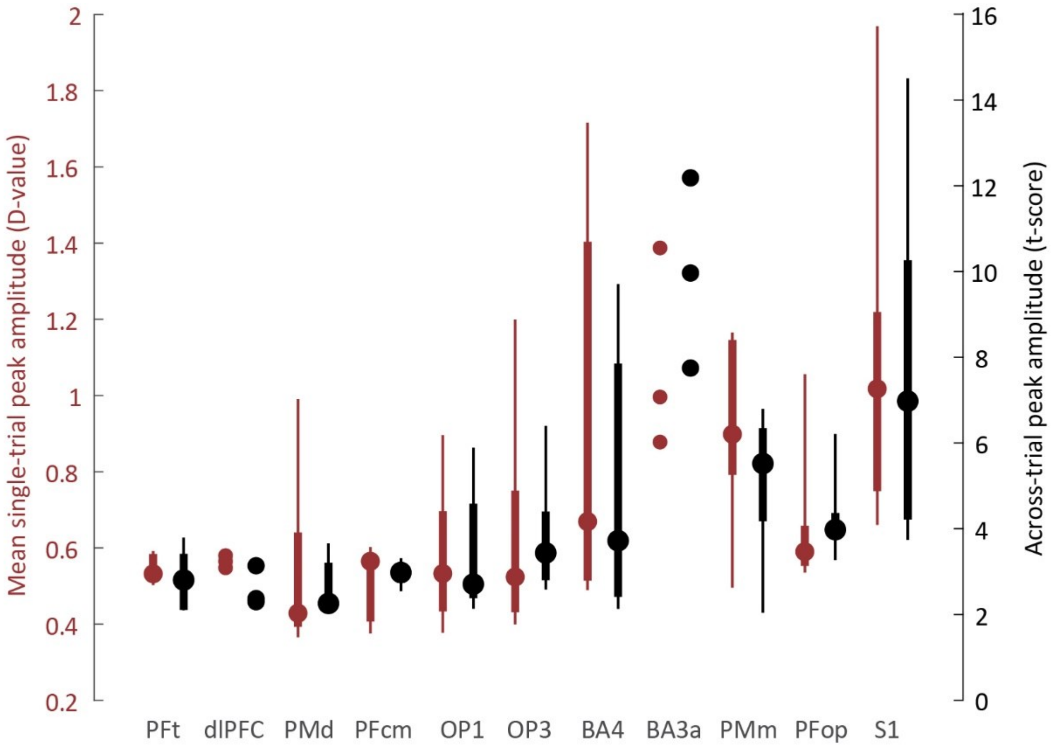

Supplementary Figure 14. Relationship between onset latency of decision brain areas and classifier amplitude for both the leave-one-out (red) and the k-fold (black) classifier results. The areas are ordered according to the rank conjunction in Figure 5a. For the leave-one-out classifiers, peak amplitudes were determined for each trial and then averaged to obtain an average peak value for every lead. These average D-value peaks are plotted in red (left y-axis). The peak t-scores across trials of the k-fold classifier results are given in black (t-scores, right y-axis). The data are represented as box plots across leads (number of leads per region is given in Supplementary Figure 3), showing the 5, 25, 50 (filled circles), 75 and 95% boundaries.

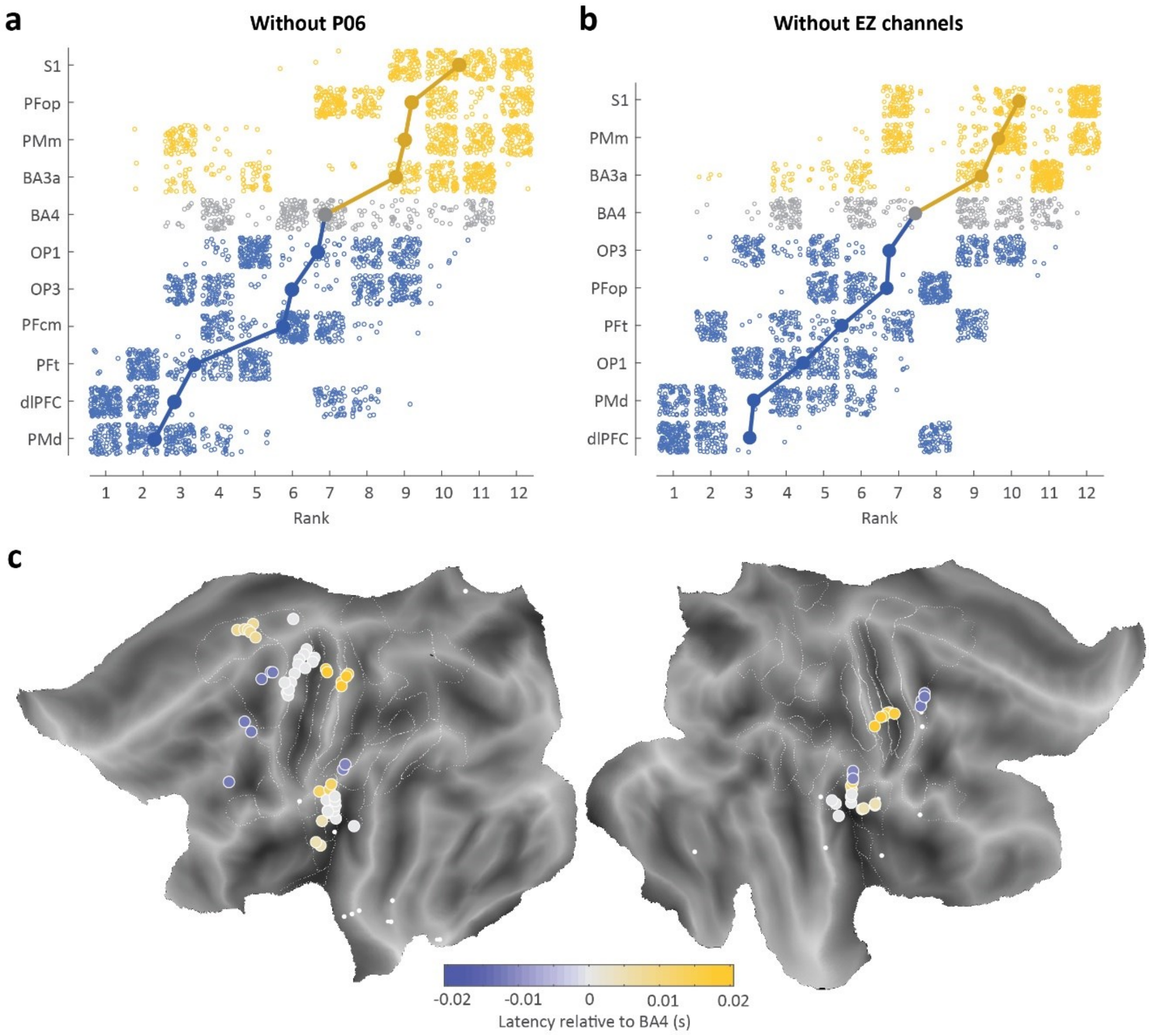

Supplementary Figure 15. Control analyses for the results presented in Figure 5. a: Rank consistency across the four analysis approaches without the data of patient 6 leads to a timeline that is almost identical to the full dataset; b: Rank consistency across the four approaches without the leads located in EZ yields slightly more noisy, but otherwise similar results to the full dataset. Note that a and b have different y-axes; c: Average onset times per brain area for all areas with high consistency (see main text). This panel shows the same data as Figure 5b, but shows the individual leads contributing to the result.

Supplementary References

[1] Spinnler, H. & Tognoni, G. Standardizzazione e taratura italiana di test neuropsicologici. *Ital. J. Neurol. Sci*. 8(Suppl), 1–120 (1987).

[2] Kaplan, E., Goodglass, H. & Weintraub, S. The Boston Naming Test. (Lea and Febiger, Philadelphia, 1983).

[3] Biancardi, A. & Stoppa, E. Il test delle Campanelle modificato: una proposta per lo studio dell'attenzione in etá evolutiva. *Psichiatr. dell'infanzia e dell'adolescenz*a 64, 73–84 (1997).

[4] Hamsher, K. D., Levin, H. S. & Benton, A. L. Facial recognition in patients with focal brain lesions. *Arch. Neurol*. 36, 837–839 (1979).

[5] Eickhoff, S. B., Stephan K.E., Mohlberg, H., Grefkes, C., Fink, G.R., Amunts, K., Zilles, K. A new SPM toolbox for combining probabilistic cytoarchitectonic maps and functional imaging data. *Neuroimage* 25, 1325–35 (2005).
