# Supplementary material for: Human stereoEEG recordings reveal network dynamics of decision-making in a rule-switching task
Source: Nat Commun. 2020 Jun 17;11:3075. doi: 10.1038/s41467-020-16854-w (PMC7300004; doi:10.1038/s41467-020-16854-w)
Supplement: Supplementary file 3 — Reporting Summary [file 41467_2020_16854_MOESM3_ESM.pdf]

nature research

Corresponding author(s): Marije ter Wal

Last updated by author(s): Mar 25, 2020

Reporting Summary

Nature Research wishes to improve the reproducibility of the work that we publish. This form provides structure for consistency and transparency in reporting. For further information on Nature Research policies, see Authors & Referees and the Editorial Policy Checklist.

Statistics

For all statistical analyses, confirm that the following items are present in the figure legend, table legend, main text, or Methods section.

n/a	Confirmed	
☐	☒	The exact sample size (n) for each experimental group/condition, given as a discrete number and unit of measurement
☒	☐	A statement on whether measurements were taken from distinct samples or whether the same sample was measured repeatedly
☐	☒	The statistical test(s) used AND whether they are one- or two-sided<br>*Only common tests should be described solely by name; describe more complex techniques in the Methods section.*
☒	☐	A description of all covariates tested
☐	☒	A description of any assumptions or corrections, such as tests of normality and adjustment for multiple comparisons
☐	☒	A full description of the statistical parameters including central tendency (e.g. means) or other basic estimates (e.g. regression coefficient) AND variation (e.g. standard deviation) or associated estimates of uncertainty (e.g. confidence intervals)
☐	☒	For null hypothesis testing, the test statistic (e.g. F, t, r) with confidence intervals, effect sizes, degrees of freedom and P value noted<br>*Give P values as exact values whenever suitable.*
☒	☐	For Bayesian analysis, information on the choice of priors and Markov chain Monte Carlo settings
☒	☐	For hierarchical and complex designs, identification of the appropriate level for tests and full reporting of outcomes
☐	☒	Estimates of effect sizes (e.g. Cohen's d, Pearson's r), indicating how they were calculated

Our web collection on statistics for biologists contains articles on many of the points above.

Software and code

Policy information about availability of computer code

Data collection	The stereo-EEG data were collected using Nihon Khoden EEG data acquisition software.
Data analysis	Most analyses were performed using MATLAB R2018a (The Mathworks, Inc.). Recording leads were localized using the 3D Slicer software (Fedorov et al., 2012; https://www.slicer.org/), Freesurfer surfaces (https://surfer.nmr.mgh.harvard.edu/) and the SPM Anatomy Toolbox for MATLAB (https://www.fz-juelich.de/inm/inm-1/DE/Forschung/_docs/SPMAnatomyToolbox/SPMAnatomyToolbox_node.html). Classifiers were trained using the MVPA-Light toolbox for MATLAB (https://github.com/treder/MVPA-Light).<br>The custom analysis functions described in Methods sections 7, 9 and 10 are available on https://github.com/marijeterwal/seq-reconstruct.

For manuscripts utilizing custom algorithms or software that are central to the research but not yet described in published literature, software must be made available to editors/reviewers. We strongly encourage code deposition in a community repository (e.g. GitHub). See the Nature Research guidelines for submitting code & software for further information.

Data

Policy information about availability of data

All manuscripts must include a data availability statement. This statement should provide the following information, where applicable:

- Accession codes, unique identifiers, or web links for publicly available datasets
- A list of figures that have associated raw data
- A description of any restrictions on data availability

All data underlying the results in the paper have been made publicly available through the following figshare repository: https://doi.org/10.6084/m9.figshare.c.4805487. The following data will be deposited: 1) the across-trial classifier performance for all leads (t-values), for all trials combined, as well as for rule, congruency and stimulus features separately and their label shuffled reference distributions; 2) the single trial classifier performance (D-values) for the significant leads; 3) the power spectra for the significant leads. Other data can be provided upon reasonable request, provided doing so does not violate data protection and consent restrictions.

Field-specific reporting

Please select the one below that is the best fit for your research. If you are not sure, read the appropriate sections before making your selection.

☒ Life sciences ☐ Behavioural & social sciences ☐ Ecological, evolutionary & environmental sciences

For a reference copy of the document with all sections, see nature.com/documents/nr-reporting-summary-flat.pdf

Life sciences study design

All studies must disclose on these points even when the disclosure is negative.

Sample size	Sample size was determined based on the electrode coverage of frontal cortex regions, as previous work in monkey (Buschman et al., 2012) suggested that frontal cortex plays an important part in the rule-switching aspect of the task. The recorded patients had a high overall coverage of frontal areas (> 300 electrodes across the six patients).
Data exclusions	Trials that showed artifacts resembling IEDs were manually rejected by an author (AP) not involved in the task design and further data analysis. The criteria for rejection were pre-established and used in previous studies by author AP (for example, Platonov et al., Communications Biology, 2018). The number of rejected trials for each patient are reported in Table S4.
Replication	We did not replicate the results in an independent group of subjects, however all brain regions of interest did have data points from at least two subjects. We did replicate the analysis of timing by determining the sequence based on both the classifier and the power spectra (Figure 4), leading to comparable outcomes and as a result, all attempts at replication were succesfull.
Randomization	All subjects performed the same task. The stimulus and rule sequences were randomized, with the rule switching after 10 to 46 trials.
Blinding	As all subjects performed the same task, so blinding was not relevant.

Reporting for specific materials, systems and methods

We require information from authors about some types of materials, experimental systems and methods used in many studies. Here, indicate whether each material, system or method listed is relevant to your study. If you are not sure if a list item applies to your research, read the appropriate section before selecting a response.

Materials & experimental systems

n/a	Involved in the study	
☒	☐	Antibodies
☒	☐	Eukaryotic cell lines
☒	☐	Palaeontology
☒	☐	Animals and other organisms
☐	☒	Human research participants
☒	☐	Clinical data

Methods

n/a	Involved in the study	
☒	☐	ChIP-seq
☒	☐	Flow cytometry
☒	☐	MRI-based neuroimaging

Human research participants

Policy information about studies involving human research participants

Population characteristics	6 subjects, of which 1 female, aged 19-44 years (mean 33). Performance on test of cognitive function and information about epilepsy diagnosis and medication are given in Tables S1 and S2.
Recruitment	Patients scheduled to undergo epilepsy monitoring using stereoEEG were asked by the clinical team whether they would be willing to take part in the study. The clinical team and the patients were made aware of the task content and overall research aims prior to the study, but not of the specific data analysis plan. The authors who collected the data were not involved in data analysis. It is therefore unlikely that (self-) selection biases affected the outcomes in the current report.
Ethics oversight	The Ethics Committee of Ospedale Niguarda-Ca' Granda (ID 7-012013-25.01.2013) approved the study.

Note that full information on the approval of the study protocol must also be provided in the manuscript.
